# Accuracy of the Estimated Prevalence of Childhood Obesity from Height and Weight Values Reported by Parents: Results of the Toyama Birth Cohort Study

**DOI:** 10.2188/jea.12.9

**Published:** 2007-11-30

**Authors:** Michikazu Sekine, Takashi Yamagami, Shimako Hamanishi, Sadanobu Kagamimori

**Affiliations:** 1 Department of Welfare Promotion and Epidemiology, Toyama Medical and Pharmaceutical University, Faculty of Medicine.; 2 Hokuriku Health Service Association.

**Keywords:** child, parent, obesity, prevalence, screening

## Abstract

The aim of the present study was to evaluate the validity of the estimated prevalence of childhood obesity from height and weight reported by parents. The subjects were 170 first-grade children (83 males and 87 females) and 206 fourth-grade children (99 males and 107 females). A questionnaire including questions on height and weight was distributed to children and completed by parents. Anthropometric measurements were conducted in the standard way. Age- and sex-specific cut-off points linked to adult overweight were employed to determine childhood obesity. The correlation and difference between reported and measured values were calculated separately for grade and gender. Correlation coefficients ranged from 0.90 to 0.96 for height, 0.95 to 0.99 for weight, and 0.86 to 0.97 for body mass index (BMI). Sensitivity and specificity, the indices predicting the presence or absence of actual obesity from reported height and weight, ranged from 83.3 to 93.3 % and 96.3 to 98.9 %, respectively. The estimated prevalence of obesity (as calculated by reported data) minus actual prevalence (as calculated by measured data) ranged from −1.2 to 1.0 %. These results indicate that height and weight reported by parents provides a reliable assessment of childhood obesity.

## INTRODUCTION

The increase in the prevalence of childhood obesity has become a major public health concern in developed countries^1^^,^^2^^)^. Because childhood obesity is associated with biological abnormality^3^^-^^5^^)^ and higher future mortality^6^^,^^7^^)^, the monitoring of trends in the prevalence of obesity and obesity prevention in childhood is required.

Many previous epidemiological studies have used self-reported height and weight to assess obesity^8^^-^^12^^)^, because the actual measurements of height and weight are not feasible and very expensive. However, because tallness and thinness are accepted as the ideal body composition, particularly in western countries^13^^,^^14^^)^, several adult studies have revealed that subjects tend to over-report their height and under-report their weight, leading to an underestimation of the prevalence of obesity^8^^,^^9^^,^^12^^)^

With regard to self-reported height and weight in children, a previous study in young adolescents aged 12 to 16 revealed that the correlation between reported and measured height and weight ranged from 0.82 to 0.91 for height and 0.87 to 0.95 for weight^15^^)^. However, a previous study in elementary schoolchildren reported that approximately 10 % of children had little idea of their height and weight values; the remainder of children tended to under-report their weight and showed no consistent tendency towards under-reporting or over-reporting their height^16^^)^. The correlation coefficients for weight were 0.90 for males and 0.84 for females. The correlation coefficients for height were 0.74 for males and 0.64 for females, indicating considerable deviation between reported and measured height. Therefore, self-reported height and weight are not reliable measures in elementary schoolchildren.

In elementary schoolchildren, information on height and weight reported by parents may be helpful in evaluating the prevalence of obesity. The present study provides data on the accuracy of height and weight values of elementary schoolchildren as reported by parents and the accuracy of the estimated prevalence of childhood obesity from the reported height and weight values.

## METHODS

We are currently conducting a birth cohort study comprising of more than 10000 children born in 1989^17^^,^^18^^)^. The initial survey was conducted in 1992. The follow-up surveys were conducted in 1996 and 1999, when the subjects were 1st grade and 4th grade in elementary school, respectively. The present study was conducted in July 2000, as a sub-study of the follow-up survey.

### Study population

The total population of this study was 447 children in the 1st and 4th grades of 4 elementary schools located in an urban district in Toyama City, Japan. Approximately 2 weeks before the anthropometric measurements, an introductory letter and a questionnaire including questions on height and weight was distributed to children through their elementary schools, for completion by parents. Parental permission for children to participate was obtained from 170 first-grade children (83 males and 87 females) and 206 fourth-grade children (99 males and 107 females). The participation rate corresponded to 84.1% of the total population. Of the participants, 352 (93.6%) children had their height and weight reported by both parents. Information on heights and weights of 21 (5.1%) children was provided by their mothers. Information on heights and weights of 5 (1.4%) children was provided by their fathers.

### Questionnaire survey

In the questionnaire survey, parents administered a questionnaire that asked “How tall is your child?” and “How much does your child weigh?” According to the provided examples for children’s height and weight, parents specified their children’s height to the nearest 0.1 cm and specified their children’s weight to the nearest 0.1 kg.

### Anthropometric measurements

Anthropometric measurements were conducted at each elementary school. The heights and weights of children were measured in their shorts. Height was measured to the nearest 0.1 centimeter without shoes, using a rigid stadiometer (TTM Stadiometer, Japan). Weight was measured to the nearest 0.1 kilogram, using a weighing scale (TBF-110 Scale, Japan). The stadiometer was checked for accuracy and the weighing scale was calibrated before the examination. Height and weight were measured twice by trained examiners and the means were used in the analysis. Interrater and intrarater correlations were above 0.98 for both height and weight.

### Definition of childhood obesity

Although several indices representing childhood obesity have been proposed, an index linked to the degree of adiposity and morbidity is ideal. Body mass index (BMI ; weight in kilograms divided by square of the height in meters) is correlated to skinfold^19^^)^ and biological abnormality^20^^,^^21^^)^ and commonly used as an index of childhood obesity in previous epidemiological studies^1^^,^^7^^,^^8^^)^. We therefore used BMI as the index of childhood obesity. There are no established cut-off points for BMI for childhood obesity. Recently, age- and sex-specific cut-off points for childhood obesity were proposed on the basis of a large international survey^22^^)^. The proposed cut-off points linked to adult cut-off points were considered to be less arbitrary. We therefore employed the proposed age- and sex-specific cut-off points that have been linked to overweight in adults.

### Definition of indices representing the validity of reported height and weight

To calculate the indices predicting the presence or absence of childhood obesity from height and weight values reported by parents, true positive, true negative, false positive, and false negative were defined in the following way: true positive: actual BMI (as calculated by measured data) and estimated BMI (as calculated by reported data) both indicated positive for obesity; false positive: actual BMI indicated negative, but estimated BMI indicated positive; false negative: actual BMI indicated positive, but estimated BMI indicated negative; true negative: actual and estimated BMI both indicated negative. The estimated prevalence was defined as prevalence of obesity as calculated by estimated BMI. The actual prevalence was defined as prevalence of obesity as calculated by actual BMI.

To evaluate the validity of the estimated prevalence of childhood obesity from reported height and weight, we calculated the following indices: sensitivity: true positive / (true positive plus false negative) × 100 (%); specificity: true negative / (true negative plus false positive) × 100 (%); false positive rate: 100 − specificity (%); false negative rate: 100 − sensitivity (%); predictive value positive: true positive / (true positive plus false positive) × 100 (%); predictive value negative: true negative / (true negative plus false negative) × 100 (%).

### Statistical analysis

Statistical analysis was performed separately for grade and gender. The differences between measured and reported values were tested by a paired-t test. The correlations of measured and reported values were assessed by Pearson’s correlation coefficients.

All statistical analysis was performed by SPSS (7.5.1.J) for Windows. A two-tailed P value of less than 0.05 was considered to be significant.

## RESULTS

Table 1 shows summary statistics by grade and gender. Both male and female genders tended to overestimate their height. The difference between reported and measured height was significant in the 1st grade female children. In contrast, no consistent trends were observed in their weight and BMI. The difference between reported and measured weight was not significant in all the children. Correlation coefficients between reported and measured data ranged from 0.90 to 0.96 for height, 0.95 to 0.99 for weight, and 0.86 to 0.97 for BMI.

**Table 1.** Summary statistics by grade and gender.

	1^st^ grade		1^st^ grade		4^st^ grade		4^st^ grade	
	male		female		male		female	
	n=83		n=87		n=99		n=107	
*Age (years old)*	6.23	(0.42)	6.29	(0.46)	9.25	(0.44)	9.21	(0.41)
*Height (cm)*								
Reported (Hr)	116.9	(5.80)	117.4	(6.95)	134.9	(5.83)	134.1	(6.85)
Measured (Hm	116.5	(5.51)	116.7	(6.49)	134.7	(5.88)	134.0	(6.48)
Difference (Hr-Hm)	0.34	(1.98)	0.76	(3.11)	0.22	(1.86)	0.08	(1.89)
95%CI	(-0.09 , 0.78)		(0.10 , 1.42)*		(-0.15 , 0.59)		(-0.28 , 0.44)	
Correlation (r)	0.94***		0.90***		0.95***		0.96***	
*Weight (kg)*								
Reported (Wr)	21.9	(4.05)	21.4	(3.59)	31.8	(6.30)	30.4	(6.76)
Measured (Wm)	22.0	(4.13)	21.2	(3.57)	31.6	(6.34)	30.5	(6.76)
Difference (Wr-Wm)	-0.11	(0.79)	0.18	(1.16)	0.27	(2.09)	-0.10	(0.94)
95%CI	(-0.28 , 0.07)		(-0.06 , 0.43)		(-0.14 , 0.69)		(-0.28 , 0.08)	
Correlation (r)	0.98***		0.95***		0.95***		0.99***	
*BMI (kg/m^2^)*								
Estimated (Be)	16.0	(2.34)	15.5	(2.05)	17.4	(2.59)	16.8	(2.54)
Actual (Ba)	16.1	(2.35)	15.5	(1.88)	17.3	(2.60)	16.9	(2.54)
Difference (Be-Ba)	-0.18	(0.72)	-0.04	(1.05)	0.10	(1.17)	-0.06	(0.61)
95%CI	(-0.32 , 0.02)		(-0.27 , 0.18)		(-0.14 , 0.33)		(-0.17 , 0.06)	
Correlation (r)	0.95***		0.86***		0.90***		0.97***	

Data are expressed as mean (SD).

Hr: reported height, Hm: measured height, r: Pearson’s correlation coefficient, Wr: reported weight, Wm: measured weight, BMI: body mass index, Be: estimated BMI as calculated by Wr(kg)/(Hr(m))^2^, Ba: actual BMI as calculated by Wm(kg)/(Hm(m))^2^. 95%CI; 95% confidence interval of the mean difference between self-reported and measured data.

Significance level: * p < 0.05, *** p < 0.001.

The indices representing the validity of the estimated prevalences of childhood obesity from height and weight values reported by parents are shown in Table 2. Sensitivity ranged from 83.3 % to 93.3 %. Specificity ranged from 96.3 to 98.9 %. False positive rate ranged from 1.1 % to 3.7 %. False negative rate ranged from 6.7 % to 16.7 %. Predictive value positive rates ranged from 84.2 % to 93.3 %. Predictive value negative rates ranged from 97.2 % to 98.6 %.The difference between the estimated and the actual prevalences of childhood obesity ranged from −1.2% to 1.0 %.

**Table 2.** Accuracy of the estimated prevalences of obesity from height and weight values reported by parents.

	1^st^ grade	1^st^ grade	4^st^ grade	4^st^ grade
	male	female	male	female
	n=83	n=87	n=99	n=107
True positive (n)	10	14	16	13
False positive (n)	1	1	3	1
False negative (n)	2	1	2	2
True negative (n)	70	71	78	91
Sensitivity (%)	83.3	93.3	88.9	86.7
Specificity (%)	98.6	98.6	96.3	98.9
False positive rate (%)	1.4	1.4	3.7	1.1
False negative rate (%)	16.7	6.7	11.1	13.3
Predictive value positive (%)	90.9	93.3	84.2	92.9
Predictive value negative (%)	97.2	98.6	97.5	97.8
Estimated prevalence (%) (Pe)	13.3	17.2	19.2	13.1
Actual prevalence (%) (Pa)	14.5	17.2	18.2	14.0
Difference (%) ( Pe-Pa )	-1.2	0.0	1.0	-0.9

Pe: the estimated prevalence of obesity as calculated by body mass index (BMI) from reported height and weight values, Pa: the actual prevalence of obesity as calculated by BMI from actual height and weight values.

## DISCUSSION

The present study indicated that height and weight values reported by parents were strongly related to actual height and weight and the difference between the actual and estimated prevalences of childhood obesity was quite small.

However, the present study has some limitations in the interpretation of the results. Firstly, because our study included a relatively small number of subjects, the number defined as obese subjects is small. This might influence sensitivity, because one misclassification corresponds to a 5.6 to 8.3 % change in sensitivity. Secondly, the interval between the questionnaire survey and periodic anthropometric measurement in elementary school might have influenced the accuracy of the reported height and weight. In Japan, anthropometric measurement is conducted in April, September, and January by elementary school nurses. Because results of the measurements are sent to parents, they know the heights and weights of their children. However, because the heights and weights of children change dramatically even in a year, height and weight values reported by parents could be inaccurate if the interval between the questionnaire survey and periodic anthropometric measurement is long. Because this study was conducted in July, approximately 3 months after the periodic anthropometric measurement, the accuracy might have been higher if it had been conducted earlier.

In this study, correlation coefficients between measured and reported height and weight values ranged from 0.90 to 0.99. In adults, previous studies have shown that correlation coefficients between measured and reported height and weight ranged from 0.96 to 0.99^23^^-^^25^^)^. In young adolescents, correlation coefficients between measured and reported height and weight ranged from 0.82 to 0.95^15^^)^. Therefore, the results in the present study were consistent with those in previous studies for adults and young adolescents.

In this study, reported height was consistently higher than measured height in all the children. These results are consistent with previous adult studies^8^^,^^9^^,^^13^^)^, in which the subjects tend to over-report their height. In contrast, self-reported height was slightly lower than measured height in young adolescents^15^^)^.

With regard to reported weight, previous studies in young adolescents^15^^)^ and sixth-graders^16^^)^ have indicated that reported weight tended to be lower than measured weight and more accurate in males than in females. Several previous studies^26^^,^^27^^)^ have reported that preadolescent and adolescent females are twice more likely to report a desire to be thinner than preadolescent and adolescent males. In contrast, no gender difference was observed in this study. The reason for the absence of a gender difference in this study may be that weight reported by parents is a more objective measure of weight in children and doesn’t reflect the desire to be thin.

Furthermore, correlation coefficients in the present study were higher than those of a previous study, in which sixth-graders reported their height and weight; correlation coefficients ranged from 0.84 to 0.90 for weight and 0.64 to 0.74 for height^16^^)^. Therefore, in elementary schoolchildren, height and weight reported by parents can be more accurate than self-reported height and weight.

As for the estimated prevalence of childhood obesity from reported data, both sensitivity and specificity were high, although sensitivity was relatively lower than specificity in the present study. In a previous study^28^^)^, estimates of the prevalence of obesity based on self-reported height and weight were 83 % for men and 89 % for women in terms of sensitivity and 96 % for men and 97 % for women in terms of specificity. Thus, the previous study also indicated relatively lower sensitivity compared to specificity. The reason for the relatively lower sensitivity may result from the over-reporting of height, which leads to the underestimation of BMI and an increase in the false negative rate in the present study.

In conclusion, height and weight values reported by parents were closely related to measured height and weight. Furthermore, the difference between the estimated and actual prevalences of childhood obesity was quite small. Although, needless to say, anthropometric measurements of children are desirable to assess childhood obesity, the heights and weights of children reported by parents may alternatively provide a reliable assessment of childhood obesity.
